# Neutrophil Extracellular Trap Production in Patients with Colorectal Cancer In Vitro

**DOI:** 10.1155/2017/4915062

**Published:** 2017-07-30

**Authors:** J. J. R. Richardson, C. Hendrickse, F. Gao-Smith, D. R. Thickett

**Affiliations:** ^1^Institute of Inflammation and Ageing, College of Medical and Dental Sciences, The University of Birmingham, Birmingham B15 2TT, UK; ^2^Department of Colorectal Surgery, Heart of England NHS Foundation Trust, Birmingham B9 5SS, UK

## Abstract

**Purpose:**

Neutrophil Extracellular Traps (NETs) are extracellular neutrophil derived DNA webs which have been implicated in cancer progression and in the development of metastases. NETs production in patients with colorectal cancer was investigated to elucidate their role and prognostic significance.

**Methods:**

Systemic neutrophils were isolated from consecutive patients with colorectal cancer and from age-matched healthy volunteers. Neutrophils were stimulated to produce NETs which were quantified by a measure of the fluorescence of the extracellular DNA. The impact of cancer location, tumour stage, and patient outcomes (complications, length of stay, and mortality) on NET production was investigated.

**Results:**

Quantification of NET formation was performed in patients with colorectal cancer (*n* = 45) and in well-matched healthy individuals (*n* = 20). Significant increases in NETs production in response to no stimulant (9,735 AFU versus 11347 AFU, *p* = 0.0209), IL-8 (8,644 AFU versus 11,915 AFU, *p* = 0.0032), and LPS (10,576 AFU versus 12,473 AFU, *p* = 0.0428) were identified in patients with colorectal cancer. A significant increase in NETs production in response to fMLP was detected in patients who developed significant postoperative complications (11,760 AFU versus 18,340 AFU, *p* = 0.0242) and who had a prolonged hospital recovery (9,008 AFU versus 12,530 AFU, *p* = 0.0476). An increase in NETs production was also observed in patients who died, but this did not reach statistical significance. Cancer location and tumour stage did not appear to affect preoperative NETs production.

**Conclusions:**

Patients with colorectal cancer have significantly increased NETs production in vitro when compared to healthy volunteers, possibly implicating them in cancer development. Adverse patient outcomes were associated with increased preoperative NETs production, which highlights them as potential therapeutic targets.

## 1. Introduction

Neutrophils function as the first line of defence against infections and are responsible for the containment and elimination of pathogens. They are prevalent at sites of tissue trauma and are the hallmark of acute inflammation [1]. Neutrophils are also appreciated to have an important role in cancer progression and dissemination [2]. It has been suggested that neutrophils are not a homogenous population of cells and may consist of protumour and antitumour subpopulations. The polarisation of neutrophils towards a protumour or antitumour phenotype may be mediated by the chemokine landscape in the tumour microenvironment [3]. Neutrophil Extracellular Traps (NETs) are extracellular neutrophil derived DNA webs which have been implicated in cancer progression and in the development of metastases. NETs have been demonstrated to trap circulating tumour cells with a subsequent increase in the gross macro metastatic disease burden, following tumour cell injection, in a murine model of infection of caecal ligation and puncture [2].

Activated neutrophils can undergo “NETosis.” This is an active form of cell death which leads to the release of decondensed chromatin into the extracellular space [4, 5]. The fibrous structures called NETs constitute a DNA backbone containing histones and neutrophil granule products (myeloperoxidase [MPO]) and cytoplasmic proteins (bactericidal/permeability-increasing protein [BPI], neutrophil elastase, cathepsin G, and lactoferrin) [6]. The exact mechanism for NET formation is not yet completely understood. It is thought that the ROS pathway is involved as both Nicotinamide Adenine Dinucleotide Phosphate Oxidase (NADPH) and MPO are required for NET formation [5, 7, 8]. The process of oxidant-dependent NET formation results in eventual cell death, which is distinct from apoptosis and necrosis [5, 9].

NETs are recognised as an effective antimicrobial mechanism, whereby microbes are trapped and exposed to high local concentrations of antimicrobials [10]. Conversely, NETs have been shown to have detrimental effects on the host with the formation of autoantibodies against chromatin and neutrophil components [11] and the adherence of platelets [12], which has implicated them in autoimmune and thromboembolic diseases, respectively.

NETs have been implicated in T-cell priming [13] and in the propagation of antitumour immune responses [14]. Conversely, and more frequently, they have been incriminated in tumour progression and tumour dissemination [15]. The role of NETs in tumour progression is poorly understood but the evidence to date proposes an association between intratumoural NET deposition and tumour progression in both experimental models and in patients with cancer [2, 14, 16]. The ability of tumours to predispose neutrophils to produce NETs has been demonstrated in murine models and various tumour types have been shown to predispose circulating neutrophils to produce NETs via NETosis [17]. The evidence supports the theory that demonstrates that primary tumours, through a systemic effect on the host, can induce an increase in peripheral blood neutrophils that are predisposed to NET formation.

It is anticipated that a greater understanding of NETs production in patients with colorectal cancer may help elucidate their role and prognostic significance. It was therefore decided to investigate NET formation in patients with colorectal cancer and to determine if differences exist in comparison to a healthy population. We hypothesised that NET production may represent a prognostic marker of dysregulated neutrophil function in patients with colorectal cancer.

## 2. Methods

The study was conducted in accordance with the ethical principles and was approved by the NRES Committee West Midlands on 30 December 2013 (13/WM/0485).

### 2.1. Recruitment of Patients

Patients undergoing an elective colorectal resection for cancer, within an established enhanced recovery programme, were identified at the Colorectal Cancer Multidisciplinary Team (MDT) meeting at the Heart of England NHS Foundation Trust between 1 January 2014 and 30 June 2015 and patients were followed up until 30 June 2016. All patients over the age of 18 who were undergoing elective colorectal resection for cancer and were able to give written informed consent were eligible to be included in the study. Patients were excluded from the study if they were had acute presentation, were pregnant, or were breast-feeding or if consent was refused.

Patient demographics, patient comorbidities, preoperative risk prediction, tumour characteristics, and operative characteristics were recorded from study participants.

Patient outcomes were collected prospectively and included the following:

1. Postoperative complications up to 30 days after surgery using predefined criteria and graded by the Clavien-Dindo classification system
2. Return to theatre within 28 days of the index procedure
3. Admission to and length of stay on the Intensive Care Unit
4. Total hospital length of stay
5. Readmissions to hospital within 30 days of the index procedure requiring a hospital stay ≥ 24 hours
6. 30-Day and 90-day mortality.

Haematological and biochemical parameters were collected prospectively from the hospital pathology system. These parameters, in conjunction with physiological indices, were used to determine a CR-POSSUM, validated, and risk-adjusted mortality prediction score.

### 2.2. Recruitment of Healthy Controls

Healthy volunteers were required to act as controls for patients. They were recruited from the Birmingham 1,000 Elders Cohort, a research cohort of healthy volunteers above the age of 60 willing to take part in medical research. To be considered healthy, participants had to be not suffering from serious debilitating acute or chronic illness. Well-controlled health problems, such as hypertension and asthma, did not preclude enrolment.

### 2.3. Sample Collection

Patients and healthy controls underwent peripheral blood tests performed by an experienced medical practitioner by peripheral venepuncture. Samples were obtained from patients on the morning of planned surgery prior to the commencement of any perioperative interventions.

### 2.4. Neutrophil Isolation

4 ml of 2% Dextran (Sigma-Aldrich, Dorset, UK) was added to 24 ml of blood and gently mixed by inversion. The solution was incubated at room temperature for 30 minutes to sediment the erythrocytes. The leucocyte-rich plasma was carefully layered on a Percoll (Sigma-Aldrich) density gradient consisting of 2.5 ml of 80% Percoll and 5 ml of 56% Percoll in a 15 ml sterile Falcon-TM tube. This was then centrifuged at 220 ×g for 20 minutes at room temperature. The neutrophils were then isolated from the 80% and 56% gradient interface. The neutrophils were suspended and subsequently washed in Phosphate-Buffered Saline (PBS; Gibco, Invitrogen, Paisley, UK). The resultant supernatant was removed and the neutrophils were resuspended in RPMI-1640 (Sigma-Aldrich) at a concentration of 1 × 10^6^/ml. The purity and the viability of the neutrophil yield were checked using Giemsa stain (Diff-Quik, Gentaur Europe, Brussels, Belgium) and Trypan-Blue exclusion, respectively. A purity of ≥95% and a viability of ≥97% were routinely achieved.

### 2.5. Quantification of Neutrophil Extracellular Trap Formation

The generation and release of NETs have been shown to be induced by a variety of internal and pathogen-derived molecular signals. These signals include chemokines such as interleukin-8 (IL-8) and lipopolysaccharide (LPS), formylated peptides such as N-formyl-methionyl-leucyl-phenylalanine (fMLP), and pharmacological agents, such as phorbol-12-myristate-13-acetate (PMA). These stimulants were used in the quantification of Neutrophil Extracellular Trap assay.

Neutrophils were resuspended in RPMI-1640 with 2 nM L-glutamine, 100 U/ml streptomycin, and 100 ug/ml penicillin (all from Sigma-Aldrich) at a concentration of 1 × 10^6^/ml. 1 × 10^5^ neutrophils, at a concentration of 1 × 10^6^/ml, were sited in 20 wells of a 96-well flat bottomed plate (Becton-Dickinson). An additional 75 *μ*l of RPMI-1640 with GPS was added to each well. The neutrophils in 4 wells were then stimulated with 25 *μ*l of each of the following stimulants (all from Sigma-Aldrich): PMA (1 mg/ml [1 : 800 then 1 : 10 dilution]), IL-8 (6.25 *μ*M [1 : 625 dilution]), LPS (1 mg/ml [1 : 1250 dilution]), and fMLP (10 mM [1 : 500 dilution]), and an additional 25 *μ*l of RPMI-1640 with GPS was added as a negative “unstimulated” control to the 4 remaining wells. The plate was then incubated for 3 hours at 37°C with 5% CO_2_. Each well was then treated with 200 units of Micrococcal Nuclease (MNase; Sigma-Aldrich) and 1 *μ*M of SYTOX Green (Gibco, Invitrogen) and incubated for 10 minutes at room temperature in the dark. This process stained and digested the extracellular DNA. The contents of each of the wells were then transferred into individual 500 *μ*l Eppendorf tubes and pelleted at 5000 rpm for 10 minutes. 160 *μ*l of the DNA containing supernatant was transferred into a black 96-well flat bottomed plate (Costar, Sigma-Aldrich) and fluorescence measured in a BioTek-Synergy-2 fluorometric plate reader (NorthStar Scientific Ltd., Leeds, UK) with a filter setting of 485 nm excitation and 530 nm emission. NETs were recorded in arbitrary fluorescent units (AFU) and the mean was calculated from the 4 wells for each stimulant.

**Figure 1:**
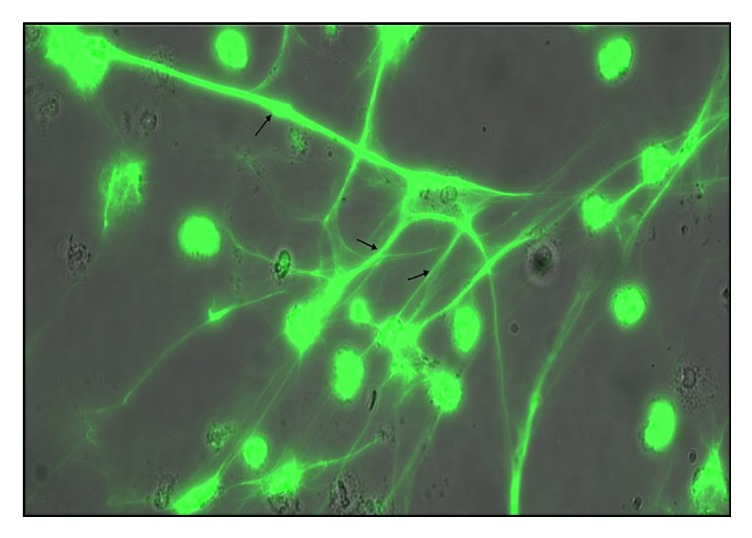
Fluorescent microscopy image of NETs (marked with arrows) following incubation with 25 *μ*l PMA (1 mg/ml [1 : 800 then 1 : 10 dilution]).

A fluorescent microscopy image of the NETs formed following incubation with 25 *μ*l PMA (1 mg/ml [1 : 800 then 1 : 10 dilution]) is shown in Figure 1. The image was created by immunofluorescence microscopy in which cells were fixed in 2% paraformaldehyde, permeabilized in 0.1% Triton x-100 (Sigma-Aldrich), and stained with 1 *μ*m SYTOX Green. Once stained, specimens were mounted in Fluoromount medium onto glass microscope slides (VWR International) and visualized using a LEICA DMI6000 B microscope (Leica Microsystems, Milton Keynes, UK) at ×20 objective. Extracellular DNA in the form of a “line” or “cloud” was used to define the presence of NETs.

### 2.6. Statistical Analysis

Statistical analysis was performed using GraphPad Prism Version 6 (La Jolla, California, USA). Categorical data was analysed using Fisher's exact tests for two variables or *χ*^2^ tests for more than two variables. Continuous data was analysed using nonparametric statistical models. Mann–Whitney *U* tests were used for independent samples for two groups. Receiver operator characteristic (ROC) curves were used to measure how well diagnostic tests distinguish between two diagnostic groups. All statistical tests performed were two-tailed and the results were considered significant when *p* < 0.05. Data is represented graphically using Box-and-Whisker plots which demonstrate the median, interquartile range, lower extreme (10%), and upper extreme (90%) of individual data sets.

## 3. Results

55 patients were identified at the MDT meeting and approached for inclusion into the study. 45 patients (81.8%) were successfully recruited into the study and were followed up for a median of 21.3 months (IQR: 16.7–23.5 months). The remaining 10 patients refused to give their consent (18.2%).

The median age of the population was 69 years (IQR: 63–75 years) and 26 patients (57.8%) were males. The median BMI was 26.75 kg/m^2^ (IQR: 22.5–28.7 kg/m^2^). 14 patients (31.1%) had two or more significant comorbidities and 16 patients (35.6%) had an ASA score of 3 or 4. The predicted mortality of the population, calculated by the CR-POSSUM, was 2.5% (IQR: 1.3–3.9%).

39 patients (86.7%) were symptomatic and the remaining 6 patients (13.3%) were identified through the NHS Bowel Cancer Screening Programme. 27 patients (60.0%) had colonic cancer, whereas 18 patients (40.0%) had rectal cancer or a cancer of the rectosigmoid junction and 11 patients (24.4%) underwent neoadjuvant therapy (10, long-course chemoradiotherapy, and 1, short-course radiotherapy) prior to surgery. 31 patients (70.5%) had early-stage disease (Dukes' A or B) versus 13 patients (29.5%) who had late-stage (node positive or metastatic) disease (Dukes' C or D).

44 patients (97.8%) underwent surgical resection. 1 patient was deemed unfit for surgery on the day of surgery and never progressed to surgical resection. 27 procedures (61.4%) were completed entirely laparoscopically and the median operative time for all operations was 174 minutes (IQR: 129–240 minutes). 16 patients (36.4%) underwent stoma formation, the majority of which were temporary ileostomies (10 patients [62.5%]) to cover a colorectal anastomosis. 17 patients (38.6%) required admission to Critical Care Unit following surgery; the majority were planned admissions (12 patients [70.6%]) and a reflection of the patients' comorbidity.

The baseline population characteristics and operative characteristics are shown in Tables 1 and 2, respectively.

22 patients (50.0%) developed a complication, although only 6 patients (13.6%) developed a significant complication (Clavien-Dindo classification ≥ 3). The median length of stay was 8 days (IQR: 5.3–10.1 days) and only 3 patients (6.8%) were readmitted up to 30 days of the index procedure. 30-Day, 90-day, and 12-month mortality was 2.3%, 4.5%, and 6.8%, respectively, with a median follow-up of 21.3 months (IQR: 16.7–23.5 months). During the entire study period, 5 patients (11.4%) died and 4 patients (9.1%) developed recurrent disease but were still alive.

Of the patients that developed complications, the most frequent were cardiorespiratory events (14) that encompassed lower respiratory tract infection (4), ischaemic events (4), arrhythmias (4), and cardiac failure (2). This was followed by mechanical/functional bowel obstruction (7) and surgical site infection (7). 3 patients (6.8%) underwent reoperation: anastomotic repair and defunctioning loop ileostomy (1), evacuation of haematoma and defunctioning ileostomy (1), and resuture of abdominal wall (1).

The patient outcomes are shown in Table 3.

Quantification of NET formation was performed in healthy individuals (*n* = 20) and in patients with colorectal cancer (*n* = 45). Populations were well matched with regard to age (67.5 versus 69.0, *p* = 0.1961) and gender (50.0% males versus 57.8% males, *p* = 0.5981). Significant increases in NET production in response to no stimulant (9,735 AFU versus 11,347 AFU, *p* = 0.0209), IL-8 (8,644 AFU versus 11,915 AFU, *p* = 0.0032), and LPS (10,576 AFU versus 12,473 AFU, *p* = 0.0428) were identified in patients with colorectal cancer. No significant differences were identified in response to PMA (42,173 AFU versus 39,238 AFU, *p* = 0.2353) or fMLP (9,566 AFU versus 12,194 AFU, *p* = 0.0868).

**Figure 2:**
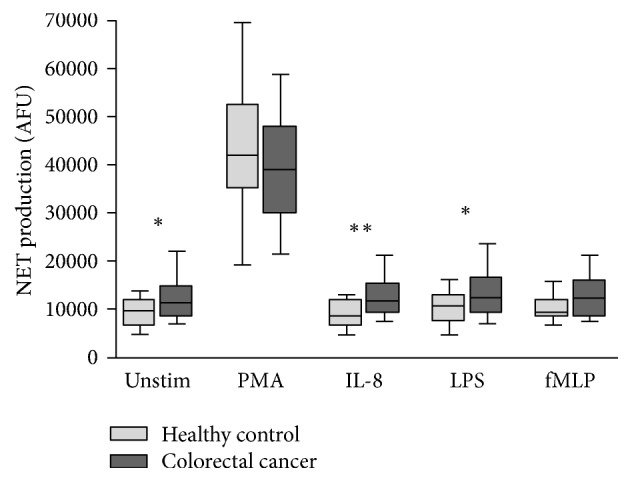
Box-and-Whisker plot (10th–90th percentile) of NET production in healthy control subjects (*n* = 20) and patients with colorectal cancer (*n* = 45) in response to no stimulant (Unstim), PMA, IL-8, LPS, and fMLP. Statistical significance, measured by Mann–Whitney *U* test, is denoted by ^*∗*^*p* < 0.05 and ^*∗∗*^*p* < 0.01.

NET production in healthy individuals and in patients with colorectal cancer is displayed in Figure 2.

The impact of cancer location and tumour stage on NET formation was investigated. No significant differences were identified in NET production when comparing rectal and colonic cancers. Similarly, no significant differences were identified in NET production when comparing early-stage (Dukes' A or B) and late-stage (Dukes' C or D) colorectal cancers.

The effect of neoadjuvant therapy, which is generally reserved for high-risk (of local disease recurrence) rectal cancer with a threatened circumferential resection margin (<1 mm), was also analysed to determine the effect on NET production. No significant difference in NET production was identified when comparing patients who received neoadjuvant therapy to those who went straight to surgery.

An evaluation of NET formation according to patient outcome was performed. The patient outcomes investigated included postoperative complications categorised into minor complications or no complications (Clavien-Dindo classification < 3) and significant complications (Clavien-Dindo classification ≥ 3), total hospital length of stay (LOS) categorised into LOS ≤ 5 days and LOS > 5 days, and mortality.

**Figure 3:**
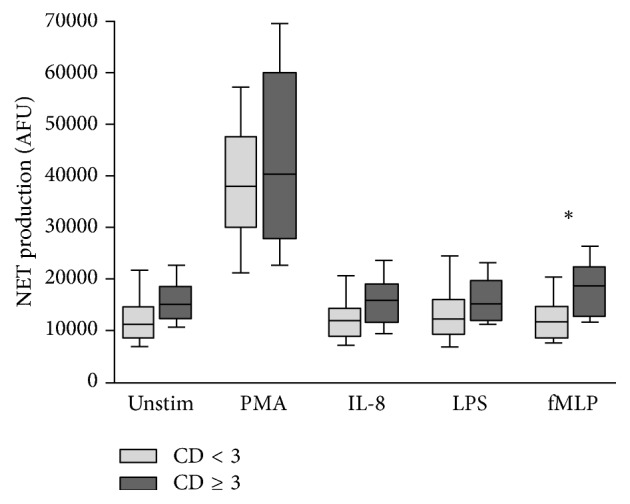
Box-and-Whisker plot (10th–90th percentile) of NET production in patients with Clavien-Dindo classification < 3 (*n* = 38) versus Clavien-Dindo classification ≥ 3 (*n* = 6) in response to no stimulant (Unstim), PMA, IL-8, LPS, and fMLP. Statistical significance, measured by Mann–Whitney *U* test, is denoted by ^*∗*^*p* < 0.05.

**Figure 4:**
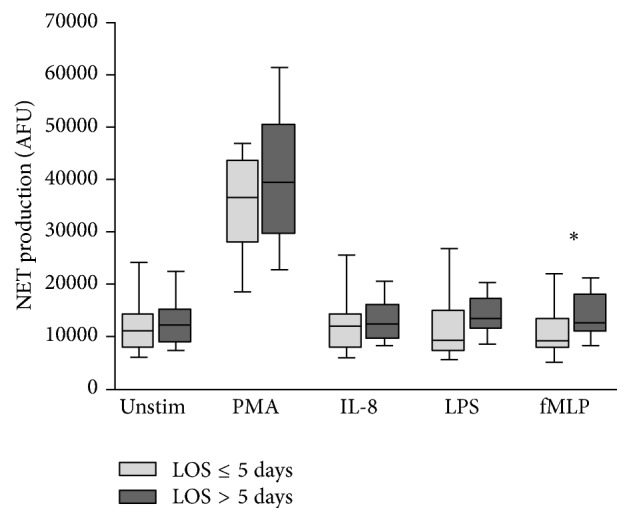
Box-and-Whisker plot (10th–90th percentile) of NET production in patients who had length of stay ≤ 5 days (*n* = 9) versus length of stay > 5 days (*n* = 35) in response to no stimulant (Unstim), PMA, IL-8, LPS, and fMLP. Statistical significance, measured by Mann–Whitney *U* test, is denoted by ^*∗*^*p* < 0.05.

**Figure 5:**
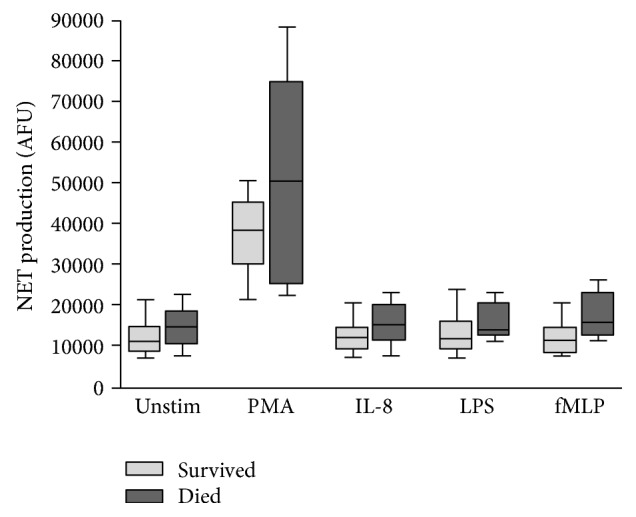
Box-and-Whisker plot (10th–90th percentile) of NET production in patients who survived (*n* = 39) versus those who died (*n* = 5) in response to no stimulant (Unstim), PMA, IL-8, LPS, and fMLP.

NET production in patients categorised by postoperative complication, LOS, and mortality is outlined in Figures 3, 4, and 5, respectively.

An increase in NET production was evident in patients who went on to develop significant complications (Clavien-Dindo classification ≥ 3) compared to patients who experienced minor complications or no complications (Clavien-Dindo classification < 3). This reached statistical significance only in response to fMLP (11,760 AFU versus 18,340 AFU, *p* = 0.0242). Similarly, an increase in NET production was evident in patients with LOS > 5 days compared to those with LOS ≤ 5 days. Again, this reached statistical significance only in response to fMLP (9,008 AFU versus 12,530 AFU, *p* = 0.0476). Increases in NET production were evident in patients who died compared to those who survived but this did not reach statistical significance. It is suggested that adverse outcomes (Clavien-Dindo classification ≥ 3, LOS > 5 days, and mortality) are all associated with increased preoperative NET production (Table 4).

**Figure 6:**
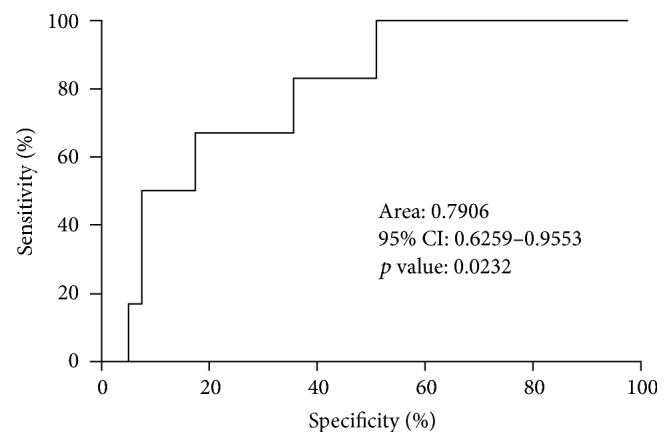
ROC curve of NET production in response to stimulation with fMLP for Clavien-Dindo classification < 3 (*n* = 38) versus Clavien-Dindo classification ≥ 3 (*n* = 6).

To assess the diagnostic ability of the quantification of NET formation assay in predicting postoperative complications, a prolonged hospital stay and mortality, ROC curve analyses were performed in patients with colorectal cancer who underwent surgical resection. When stimulated with fMLP, the area under the ROC curve (AUC) values for the development of a significant complication (Clavien-Dindo classification ≥ 3), LOS > 5 days, and mortality were 0.7906 (95% CI = 0.6259–0.9553, *p* = 0.0232), 0.6851 (95% CI = 0.5049–0.8652, *p* = 0.0463), and 0.7700 (95% CI = 0.6018–0.9382, *p* = 0.0512), respectively. The ROC curve analysis of NET production in response to stimulation with fMLP for the development of significant postoperative complications (Clavien-Dindo classification ≥ 3) is displayed in Figure 6.

## 4. Discussion

The experiments performed in this study reveal the novel finding of a significant increase in NET production in vitro in patients with colorectal cancer compared to a well-matched cohort of healthy individuals. The experimental findings support the current evidence that NETs are likely to be involved in cancer development.

It has been demonstrated, in experimental and animal studies, that neutrophils play a vital role in the development of metastases, where neutrophils facilitate circulating tumour cell adhesion to both pulmonary and hepatic endothelial surfaces [2, 18–21]. It is thought that the direct contact of circulating tumour cells and neutrophils is an important precursor to the development of metastatic disease [19, 20]. It is theorised that NETs may act within the primary tumour and promote tumour progression and dissemination, although the antimicrobial proteins and peptides associated with NETs, including Matrix Metalloproteinase-9 (MMP9), neutrophil elastase, and cathepsin G, have been implicated in tumour progression without specific reference to NETs [15]. NETs are thought to expose tumour cells to high concentrations of biologically active proteins which favour tumour proliferation and inhibit tumour cell apoptosis. It is also suggested that NETs support the dissemination of tumour cells from the primary tumour and promote early adhesive events and increase sequestration of malignant cells in end organs [19, 20].

It is also proposed that primary tumours can facilitate NET production in circulating neutrophils. This effect has been attributed, in part, to granulocyte colony-stimulating factor (G-CSF) production by tumours [22]. In addition, TNF-*α* and IL-8 have been shown to facilitate NET formation and these cytokines are highly expressed by a number of tumour types, including colorectal cancer [18, 22–24]. The findings of this study provide additional support to this theory.

Despite the accumulating evidence that suggests an association between NETs and tumour progression, the experimental evidence outlined in this study revealed no significant differences in NET production in patients with more advanced disease when patients with early-stage and late-stage colorectal cancers were compared. In addition, no significant differences were identified in NET production in patients treated with neoadjuvant therapy compared to patients who went straight to surgery or when comparing cancer locations.

The systemic inflammatory response can be assessed by examining changes in concentrations of circulating acute phase proteins, such as C-reactive protein (CRP), serum cytokines (tumour necrosis factor-*α* [TNF-*α*], interleukin-6 [IL-6], IL-8, and IL-10), and low levels of circulating albumin [25, 26]. Preoperatively, these factors have been demonstrated to be stage-independent prognostic factors in many cancer types [27–31]. The cellular components of the systemic inflammatory response, such as neutrophils, lymphocytes, monocytes, and platelets, have all been reported to have prognostic value in patients with cancer [32–36]. Preoperative changes in circulating white blood cells and, in particular, the neutrophil-lymphocyte ratio (NLR) have been used to predict overall and cancer-specific survival in numerous solid organ malignancies, including colorectal cancer [37].

Cancer-associated inflammation, both in the systemic circulation and in the tumour microenvironment, is now widely recognised to be a key determinant of disease progression and survival in cancer. A chronic dysregulation of the immune system may account for the persistently elevated systemic inflammatory response, either as a consequence of its activation by micro metastases or as a result of disease which induces tissue injury [38, 39]. It is now established that the systemic inflammatory response to a tumour is a negative prognostic factor in primary operable [40] and metastatic colorectal cancer [41–45]. It is also known that infectious complications in patients with cancer are associated with adverse oncological outcomes and an increased mortality as a consequence of metastatic disease [46–50].

The analysis performed in this study suggests that adverse patient outcomes (postoperative complications, prolonged hospital recovery, and mortality) are associated with increased preoperative NET production (to all stimulants), which indicates increased neutrophil activation, conceivably as a result of cancer-associated inflammation. Significant increases in NET production in response to stimulation with fMLP were demonstrated in patients who developed a postoperative complication (Clavien-Dindo classification ≥ 3) and in patients who had prolonged hospital stay (LOS > 5 days). ROC curve analyses were also able to distinguish between patients who developed postoperative complications and had a prolonged hospital stay with significance. NETs production, particularly in response to stimulation with fMLP, therefore has potential prognostic significance and further investigation into their predictive value is justified. fMLP stimulation of neutrophils activates a wide variety of intracellular signaling pathways mediated by phospholipase C (PLC), phospholipase D (PLD), phospholipase A_2_ (PLA_2_), phosphatidylinositol 3-kinase (PI3K), and mitogen-activated protein kinases (MAPKs) to induce various cellular functions [51]. Both PLC and PLD have been reported to be involved in superoxide generation and degranulation of neutrophils [52, 53] and this may explain the experimental findings observed in this analysis.

A dysregulated neutrophil function in patients with colorectal cancer may conceivably be attenuated by perioperative immune-modulatory therapies. These therapies aim to suppress nonspecific systemic inflammation and maintain an effective antitumour, cell-mediated immunity of the host which may assist the clearance of circulating tumour cells and the development of occult metastases [54]. Accumulating evidence suggests that simple immune-modulatory strategies (anti-inflammatory agents [nonsteroidal anti-inflammatory drugs] or immune-modulatory therapies [corticosteroids and HMG-CoA reductase inhibitors (statins)]) could be safely implemented in the perioperative period [55]. There is increasing evidence that immune-modulatory therapies are able to modulate neutrophil function. In particular, statins have been demonstrated to reduce the neutrophil infiltrate following LPS challenge in bronchoalveolar lavage fluid taken from patients with sepsis [56]. Statins have also been shown to increase NET formation in healthy individuals with the consequent elimination of bacteria in vitro [57]. Furthermore, during bacterial pneumonia-associated sepsis, NET production is suppressed with improvements occurring during sepsis resolution [58]. It has been proposed that neutrophil functional defects may be restored by the administration of statins [58]. NETs could therefore represent potential therapeutic targets and modulation of neutrophil dysregulation, as a consequence of cancer-associated systemic inflammation, may be possible.

This experimental study has a number of limitations. Firstly, the population evaluated was small and heterogeneous with regard to patient demographics, patient comorbidities, tumour characteristics, and operative characteristics, making interpretation of the results difficult and limiting the generalisability of the study's findings. Secondly, comparative analyses stratifying patients according to cancer location, cancer stage, and patient outcomes are subject to misinterpretation as potential confounding variables were not accounted for. Thirdly, the number of adverse patient outcomes in the study population was low, making interpretation of patient outcomes challenging. Fourthly, in vitro experiments performed to assess neutrophil function were conducted outside of the biological context and consequently there are challenges in extrapolating the results and it must be acknowledged that they cannot be readily transposed to and predict the reaction of the entire organism in vivo. Lastly, in healthy controls and in patients with colorectal cancer, significant increases in NET production were apparent when comparing unstimulated neutrophils with neutrophils stimulated with PMA and LPS. No significant differences were evident when comparing unstimulated neutrophils with neutrophils stimulated with IL-8 or fMLP. This may represent inadvertent neutrophil activation when processing unstimulated neutrophils or failure of induction of NET formation in response to stimulation with IL-8 and fMLP.

## 5. Conclusion

NETs have been incriminated in tumour progression and dissemination and it is suggested that tumours may predispose circulating neutrophils to produce NETs. The experimental findings revealed significantly increased NET production in patients with colorectal cancer when compared to a healthy control population and this contributes to the evidence that NETs are possibly implicated in cancer development. It has also been demonstrated that adverse patient outcomes, that is, postoperative complications, prolonged hospital stay, and mortality, were all associated with increased preoperative NET production. It is therefore conceivable that NET production may play a pathophysiological role for the development of adverse outcomes. NETs represent potential therapeutic targets and merit further investigation in the context of colorectal cancer.

## Figures and Tables

**Table 1:** Population characteristics.

Patient demographics	*N* = 45
Age, median (IQR)	69.0 (63.0–75.0)
Gender, *n* (%), M/F	26/19 (57.8/42.2)
BMI, median (IQR), kg/m^2^	26.8 (22.5–28.7)
Smoking status, *n* (%), 1/2/3^a^	9/9/27 (20.0/20.0/40.0)
ASA, *n* (%), 1/2/3/4	4/25/14/2 (8.9/55.6/31.1/4.4)
Comorbidity, *n* (%), 0/1/2/≥3	10/21/10/4 (22.2/46.7/22.2/8.9)
Medications, *n*, 1/2/3/4^b^	21/9/11/7
CR-POSSUM (predicted mortality [%]), median (IQR)	2.5 (1.3–3.9)

Presentation	*N* = 45

Presentation, *n* (%), 1/2^c^	39/6 (86.7/13.3)
Cancer location, *n* (%), 1/2^d^	27/18 (60.0/40.0)

Tumour characteristics	*N* = 44

Neoadjuvant therapy, *n* (%), y	11 (24.4)
T-Stage, *n* (%), 0/1/2/3/4	2/2/9/22/9 (4.5/4.5/20.5/50.0/20.5)
N-Stage, *n* (%), 0/1/2	32/8/4 (72.7/18.2/9.1)
M-Stage, *n* (%), 0/1	42/2 (95.5/4.5)
Dukes' stage, *n* (%), A/B/C/D	9/22/11/2 (20.5/50.0/25.0/4.5)
Differentiation, *n* (%), 1/2^e^	39/5 (88.6/11.4)
Extramural venous invasion, *n* (%), y	14 (32.6)

y: yes; 1/2/3^a^: active/former/never; 1/2/3/4^b^: antihypertensive/*β*-antagonist/antiplatelet/oral hypoglycaemic; 1/2^c^: symptomatic/screened; 1/2^d^: colon/rectosigmoid junction and rectum; 1/2^e^: well and moderately differentiated/poorly differentiated.

**Table 2:** Operative characteristics.

Operative characteristics	*N* = 44
Operation type, *n* (%), 1/2^a^	25/19 (56.8/43.2)
Operation technique, *n* (%), 1/2^b^	27/17 (61.4/38.6)
Stoma formation, *n* (%), y	16 (36.4)
Length of operation (minutes), median (IQR)	174 (129–240)
Postoperative level of care, *n* (%), 1/2^c^	27/17 (61.4/38.6)

y: yes; 1/2^a^: segmental/rectal; 1/2^b^: laparoscopic/open and laparoscopic converted to open; 1/2^c^: ward based care/critical care.

**Table 3:** Patient outcomes.

Patient outcomes		*N* = 44
Complication, *n* (%), y		22 (50.0)
Clavien-Dindo classification, *n* (%), I/II/III/IV/V		12/4/2/2/2 (27.3/9.1/4.5/4.5/4.5)
Reoperation, *n* (%)		3 (6.8)
Critical care admission, *n* (%), y		19 (43.2)
Total LOS, median (range, IQR)		8.0 (4.0–67.0, 5.3–10.0)
Readmission, *n* (%), y		3 (6.8)
Disease recurrence, *n* (%)		4 (9.1)
Mortality, *n* (%), y		
	30-Day	1 (2.3)
	90-Day	2 (4.5)
	12-Month	3 (6.8)

y: yes.

**Table 4:** Stratified results of quantification of NET formation assay.

	NET production (AFU), median (IQR)		
Stimulant	Healthy control (*n* = 20)	Colorectal cancer (*n* = 45)	*p* value

Unstimulated	9735 (6429–11840)	11347 (4927–8341)	**0.0209**
PMA	42173 (35243–52315)	39238 (29497–47988)	0.2353
IL-8	8644 (6098–12304)	11915 (9031–15358)	**0.0032**
LPS	10576 (7366–12946)	12473 (9381–16542)	**0.0428**
fMLP	9566 (8345–11973)	12194 (8602–15991)	0.0868

Stimulant	Rectal cancer (*n* = 18)	Colonic cancer (*n* = 27)	*p* value

Unstimulated	10190 (8057–14580)	12140 (9980–1499)	0.2711
PMA	39490 (29050–49190)	38100 (29370–48840)	0.8078
IL-8	11080 (8245–15610)	11960 (9998–15350)	0.4655
LPS	11600 (8515–15580)	12730 (11210–18640)	0.2813
fMLP	10520 (8384–14910)	12440 (9203–16850)	0.4241

Stimulant	Early stage (Dukes' A/B) (*n* = 32)	Late stage (Dukes' C/D) (*n* = 13)	*p* value

Unstimulated	11340 (8862–14880)	11350 (7476–15150)	0.7353
PMA	39490 (29640–47200)	38100 (27730–48530)	0.8314
IL-8	11990 (9574–15750)	10320 (7790–14820)	0.3105
LPS	12600 (9783–16280)	11540 (9106–17270)	0.6795
fMLP	12200 (8500–16050)	11780 (8624–14840)	0.6432

Stimulant	Neoadjuvant therapy (*n* = 11)	Straight to surgery (*n* = 34)	*p* value

Unstimulated	9039 (7507–13230)	11780 (9031–15460)	0.1577
PMA	34830 (27140–41590)	40330 (29560–49070)	0.2002
IL-8	10320 (8306–15190)	12010 (9349–15980)	0.2295
LPS	13900 (8323–14930)	12380 (10070–17120)	0.4516
fMLP	11400 (8339–15870)	12330 (8704–17200)	0.4205

Stimulant	Clavien-Dindo < 3 (*n* = 38)	Clavien-Dindo ≥ 3 (*n* = 6)	*p* value

Unstimulated	11090 (8240–14670)	14780 (12630–18330)	0.0639
PMA	38100 (29620–47760)	40330 (27690–60140)	0.6522
IL-8	11820 (8517–14300)	15840 (11300–19070)	0.0796
LPS	12290 (8858–15750)	15180 (11620–19710)	0.2359
fMLP	11760 (8339–14510)	18340 (12330–22400)	**0.0242**

Stimulant	LOS ≤ 5 days (*n* = 15)	LOS > 5 days (*n* = 29)	*p* value

Unstimulated	11090 (7507–14200)	12140 (8879–15260)	0.3220
PMA	36430 (27510–43650)	39740 (29500–50280)	0.2760
IL-8	11840 (7935–14200)	12250 (9261–6443)	0.4576
LPS	9408 (6915–15170)	13180 (11250–17270)	0.1812
fMLP	9008 (7599–13290)	12530 (10690–17940)	**0.0476**

Stimulant	Survived (*n* = 40)	Died (*n* = 5)	*p* value

Unstimulated	11180 (8301–14640)	14760 (10340–18880)	0.2707
PMA	38670 (29640–45400)	50810 (25310–74680)	0.3033
IL-8	11830 (9004–14970)	15370 (10960–20510)	0.1644
LPS	12290 (8982–16280)	13900 (12360–20780)	0.2405
fMLP	11680 (8354–14530)	16110 (12310–23420	0.0533

## References

[1] Borregaard N. (2010). Neutrophils, from Marrow to Microbes. *Immunity*.

[2] Cools-Lartigue J., Spicer J., McDonald B. (2013). Neutrophil extracellular traps sequester circulating tumor cells and promote metastasis. *Journal of Clinical Investigation*.

[3] Sagiv J. Y., Michaeli J., Assi S. (2015). Phenotypic diversity and plasticity in circulating neutrophil subpopulations in cancer. *Cell Reports*.

[4] Brinkmann V., Reichard U., Goosmann C. (2004). Neutrophil extracellular traps kill bacteria. *Science*.

[5] Fuchs T. A., Abed U., Goosmann C. (2007). Novel cell death program leads to neutrophil extracellular traps. *The Journal of Cell Biology*.

[6] Urban C. F., Ermert D., Schmid M. (2009). Neutrophil extracellular traps contain calprotectin, a cytosolic protein complex involved in host defense against Candida albicans. *PLoS Pathogens*.

[7] Patel S., Kumar S., Jyoti A. (2010). Nitric oxide donors release extracellular traps from human neutrophils by augmenting free radical generation. *Nitric Oxide*.

[8] Metzler K. D., Fuchs T. A., Nauseef W. M. (2011). Myeloperoxidase is required for neutrophil extracellular trap formation: implications for innate immunity. *Blood*.

[9] Steinberg B. E., Grinstein S. (2007). Unconventional roles of the NADPH oxidase: signaling, ion homeostasis, and cell death.. *Science's STKE : signal transduction knowledge environment*.

[10] Papayannopoulos V., Zychlinsky A. (2009). NETs: a new strategy for using old weapons. *Trends in Immunology*.

[11] Hakkim A., Furnrohr B. G., Amann K. (2010). Impairment of neutrophil extracellular trap degradation is associated with lupus nephritis. *Proceedings of the National Academy of Sciences of the United States*.

[12] Fuchs T. A., Brill A., Duerschmied D. (2010). Extracellular DNA traps promote thrombosis. *Proceedings of the National Academy of Sciences of the United States of America*.

[13] Tillack K., Breiden P., Martin R., Sospedra M. (2012). T lymphocyte priming by neutrophil extracellular traps links innate and adaptive immune responses. *Journal of Immunology*.

[14] Berger-Achituv S., Brinkmann V., Abed U. A. (2013). A proposed role for neutrophil extracellular traps in cancer immunoediting. *Frontiers in Immunology*.

[15] Cools-Lartigue J., Spicer J., Najmeh S., Ferri L. (2014). Neutrophil extracellular traps in cancer progression. *Cellular and Molecular Life Sciences*.

[16] Sangaletti S., Tripodo C., Vitali C. (2014). Defective stromal remodeling and neutrophil extracellular traps in lymphoid tissues favor the transition from autoimmunity to lymphoma. *Cancer Discovery*.

[17] Demers M., Krause D. S., Schatzberg D. (2012). Cancers predispose neutrophils to release extracellular DNA traps that contribute to cancer-associated thrombosis. *Proceedings of the National Academy of Sciences of the United States of America*.

[18] Huh S. J., Liang S., Sharma A., Dong C., Robertson G. P. (2010). Transiently entrapped circulating tumor cells interact with neutrophils to facilitate lung metastasis development. *Cancer Research*.

[19] McDonald B., Spicer J., Giannais B., Fallavollita L., Brodt P., Ferri L. E. (2009). Systemic inflammation increases cancer cell adhesion to hepatic sinusoids by neutrophil mediated mechanisms. *International Journal of Cancer*.

[20] Spicer J. D., McDonald B., Cools-Lartigue J. J. (2012). Neutrophils promote liver metastasis via Mac-1-mediated interactions with circulating tumor cells. *Cancer Research*.

[21] Liang S., Fu C., Wagner D. (2008). Two-dimensional kinetics of beta 2-integrin and ICAM-1 bindings between neutrophils and melanoma cells in a shear flow. *American Journal of Physiology*.

[22] Colotta F., Re F., Polentarutti N., Sozzani S., Mantovani A. (1992). Modulation of granulocyte survival and programmed cell death by cytokines and bacterial products. *Blood*.

[23] Phillipson M., Kubes P. (2011). The neutrophil in vascular inflammation. *Nature Medicine*.

[24] Al Obeed O. A., Alkhayal K. A., Al Sheikh A. (2014). Increased expression of tumor necrosis factor-*α* is associated with advanced colorectal cancer stages. *World journal of gastroenterology*.

[25] Gabay C., Kushner I. (1999). Acute-phase proteins and other systemic responses to inflammation. *The New England Journal of Medicine*.

[26] McMillan D. C., Elahi M. M., Sattar N., Angerson W. J., Johnstone J., McArdle C. S. (2001). Measurement of the systemic inflammatory response predicts cancer-specific and non-cancer survival in patients with cancer. *Nutrition and Cancer*.

[27] Heys S. D., Walker L. G., Deehan D. J., Eremin O. E. (1998). Serum albumin: a prognostic indicator in patients with colorectal cancer. *Journal of the Royal College of Surgeons of Edinburgh*.

[28] Nozoe T., Matsumata T., Kitamura M., Sugimachi K. (1998). Significance of preoperative elevation of serum C-reactive protein as an indicator for prognosis in colorectal cancer. *The American Journal of Surgery*.

[29] Longo W. E., Virgo K. S., Johnson F. E. (2000). Risk factors for morbidity and mortality after colectomy for colon cancer. *Diseases of the Colon & Rectum*.

[30] Nielsen H. J., Christensen I. J., Sorensen S. (2000). Preoperative plasma plasminogen activator inhibitor type-1 and serum C-reactive protein levels in patients with colorectal cancer. *The RANX05 Colorectal Cancer Study Group*.

[31] McMillan D. C., Canna K., McArdle C. S. (2003). Systemic inflammatory response predicts survival following curative resection of colorectal cancer. *British Journal of Surgery*.

[32] Riesco A. (1970). Five‐year cancer cure: Relation to total amount of peripheral lymphocytes and neutrophils. *Cancer*.

[33] Bruckner H. W., Lavin P. T., Plaxe S. C., Storch J. A., Livstone E. M. (1982). Absolute Granulocyte, Lymphocyte, and Monocyte Counts: Useful Determinants of Prognosis for Patients With Metastatic Cancer of the Stomach. *JAMA: The Journal of the American Medical Association*.

[34] Viganó A., Bruera E., Jhangri G. S., Newman S. C., Fields A. L., Suarez-Almazor M. E. (2000). Clinical survival predictors in patients with advanced cancer. *Archives of Internal Medicine*.

[35] Maltoni M., Caraceni A., Brunelli C. (2005). Prognostic factors in advanced cancer patients: Evidence-based clinical recommendations - A study by the steering committee of the european association for palliative care. *Journal of Clinical Oncology*.

[36] Hauser C. A., Stockler M. R., Tattersall M. H. N. (2006). Prognostic factors in patients with recently diagnosed incurable cancer: A systematic review. *Supportive Care in Cancer*.

[37] Walsh S. R., Cook E. J., Goulder F., Justin T. A., Keeling N. J. (2005). Neutrophil-lymphocyte ratio as a prognostic factor in colorectal cancer. *Journal of Surgical Oncology*.

[38] Kantola T., Klintrup K., Väyrynen J. P. (2012). Stage-dependent alterations of the serum cytokine pattern in colorectal carcinoma. *British Journal of Cancer*.

[39] Guthrie G., McMillan D. C. (2013). Comment on 'Stage-dependent alterations of the serum cytokine pattern in colorectal carcinoma'. *British Journal of Cancer*.

[40] Roxburgh C. S. D., McMillan D. C. (2010). Role of systemic inflammatory response in predicting survival in patients with primary operable cancer. *Future Oncology*.

[41] Leitch E. F., Chakrabarti M., Crozier J. E. M. (2007). Comparison of the prognostic value of selected markers of the systemic inflammatory response in patients with colorectal cancer. *British Journal of Cancer*.

[42] Read J. A., Choy S. T. B., Beale P. J., Clarke S. J. (2006). Evaluation of nutritional and inflammatory status of advanced colorectal cancer patients and its correlation with survival. *Nutrition and Cancer*.

[43] Malik H. Z., Prasad K. R., Halazun K. J. (2007). Preoperative prognostic score for predicting survival after hepatic resection for colorectal liver metastases. *Annals of Surgery*.

[44] Ishizuka M., Nagata H., Takagi K., Kubota K. (2009). Influence of inflammation-based prognostic score on mortality of patients undergoing chemotherapy for far advanced or recurrent unresectable colorectal cancer. *Annals of Surgery*.

[45] Sharma R., Zucknick M., London R., Kacevska M., Liddle C., Clarke S. J. (2008). Systemic inflammatory response predicts prognosis in patients with advanced-stage colorectal cancer. *Clinical Colorectal Cancer*.

[46] Ohtsuka T. (2009). Infectious complications after gastric cancer surgery accelerate a rapid hepatic recurrence. *Hepato-Gastroenterology*.

[47] Matsuo K., Prather C. P., Ahn E. H. (2012). Significance of perioperative infection in survival of patients with ovarian cancer. *International Journal of Gynecological Cancer*.

[48] Colotta F., Allavena P., Sica A., Garlanda C., Mantovani A. (2009). Cancer-related inflammation, the seventh hallmark of cancer: links to genetic instability. *Carcinogenesis*.

[49] Farid S. G., Aldouri A., Morris-Stiff G. (2010). Correlation between postoperative infective complications and long-term outcomes after hepatic resection for colorectal liver metastasis. *Annals of Surgery*.

[50] Andalib A., Ramana-Kumar A. V., Bartlett G., Franco E. L., Ferri L. E. (2013). Influence of postoperative infectious complications on long-term survival of lung cancer patients: A population-based cohort study. *Journal of Thoracic Oncology*.

[51] Selvatici R., Falzarano S., Mollica A., Spisani S. (2006). Signal transduction pathways triggered by selective formylpeptide analogues in human neutrophils. *European Journal of Pharmacology*.

[52] Smith R. J., Sam L. M. (1990). Receptor-coupled signal transduction in human polymorphonuclear neutrophils: effects of a novel inhibitor of phospholipase C-dependent processes on cell responsiveness. *Journal of Pharmacology and Experimental Therapeutics*.

[53] Azuma Y., Kosaka K., Kashimata M. (2007). Phospholipase D-dependent and -independent p38MAPK activation pathways are required for superoxide production and chemotactic induction, respectively, in rat neutrophils stimulated by fMLP. *European Journal of Pharmacology*.

[54] Roxburgh C. S., Horgan P. G., McMillan D. C. (2013). The perioperative immune/inflammatory insult in cancer surgery: Time for intervention?. *OncoImmunology*.

[55] Roxburgh C. S. D., McMillan D. C. (2014). Cancer and systemic inflammation: treat the tumour and treat the host. *British Journal of Cancer*.

[56] Shyamsundar M., McKeown S. T. W., O'Kane C. M. (2009). Simvastatin decreases lipopolysaccharide-induced pulmonary inflammation in healthy volunteers. *American Journal of Respiratory and Critical Care Medicine*.

[57] Chow O. A., von Köckritz-Blickwede M., Bright A. T. (2010). Statins enhance formation of phagocyte extracellular traps. *Cell Host and Microbe*.

[58] Greenwood H., Patel J., Mahida R. (2014). Simvastatin to modify neutrophil function in older patients with septic pneumonia (SNOOPI): Study protocol for a randomised placebo-controlled trial. *Trials*.

